# Cocooning: Umwelt und Geschlecht. Einleitung

**DOI:** 10.1007/s00048-020-00284-2

**Published:** 2020-12-01

**Authors:** Susanne Schmidt, Lisa Malich

**Affiliations:** 1 Institut für Geschichtswissenschaften, Lehrstuhl für Wissenschaftsgeschichte, Humboldt-Universität zu Berlin, Unter den Linden 6, 10099 Berlin, Deutschland; 2 Institut für Medizingeschichte und Wissenschaftsforschung, Universität zu Lübeck, Königsstr. 42, 23552 Lübeck, Deutschland

Die gesamte Metaphorik des Milieus, der Umgebung, der Umwelt, so schreibt der Philologe Leo Spitzer am Ende seiner Begriffsgeschichte „Milieu and Ambiance“ (1942), drücke das fundamentale Bedürfnis des Menschen nach Geborgenheit und Sicherheit aus. Im Bild der Hülle oder Eierschale, des Gefäßes („receptacle“) und der Umarmung käme dies wieder und wieder zum Ausdruck. Nicht zufällig verwendet der renommierte Stilkritiker die Formulierung „*idée-mère*“ (Ur-Idee), handle es sich doch um eine Projektion des „Gefühls des Kindes in seiner Hülle, geschützt im Bauch seiner Mutter“.^1^ Für Spitzer symbolisierte der Mutterleib jenes hegende, „warme“ Umweltverständnis, das er deterministischen Milieubegriffen entgegenstellte. Dabei deckt seine Bemerkung nicht nur ein grundlegendes Denkmuster auf, sondern verweist zugleich auch auf eine Leerstelle, die den Ausgangspunkt der hier versammelten Beiträge bildet: Vorstellungen von Atmosphäre und Weiblichkeit sind aufs Engste verknüpft, Umwelt ist weiblich codiert. Statt dies zur anthropologischen Konstante zu erklären, historisieren die hier versammelten Beiträge den Zusammenhang von Umwelt und Geschlecht. Sie wenden sich der Frage zu, auf welche Weise Frauen für die Herstellung, gar Verkörperung der Umwelt verantwortlich gemacht wurden.

Ökologie und *care* sind nicht getrennt zu denken: Die Gesamtheit der Wechselbeziehungen zwischen Lebewesen und ihren Umwelten ist stets an Tätigkeiten der Fürsorge gebunden. Damit bedingen die gegenwärtige ökologische Krise und die „crisis of care“ einander, genauso wie die Produktion von der Reproduktion, das Büro und die Fabrik von der Familie abhängen (Fraser 2016). Doch in der Forschung wurde die konstitutive Beziehung von „Umgebungswissen“ (Wessely 2013) und Geschlecht bisher kaum beleuchtet. Die Umgebungen der alltäglichen Lebenswelt, der kulturellen Nahbereiche oder der humanwissenschaftlichen Disziplinen – die Umwelten des Zuhauses, des Kinder- oder Krankenbetts ebenso wie der emotional-psychischen Beziehungen – finden selten Berücksichtigung. Gerade diese Umwelten sind jedoch, so die zentrale These des Themenhefts, weiblich codiert und bedürfen einer genaueren Reflexion.

Wissenschafts-, medizin- und technikhistorische Beiträge zu Umweltthemen haben sich bisher vor allem mit zwei Aspekten befasst: erstens mit auf den individuellen Körper zentrierten Konzepten des „inneren“ Milieus, etwa in Endokrinologie, Genetik oder Toxikologie, und zweitens mit Vorstellungen der Umwelt als Teil einer kultiviert-natürlichen Außenwelt, zum Beispiel in Ökologie oder Botanik.^2^ Dabei waren Arbeiten zum zweiten Themenspektrum in den 1980er und 1990er Jahren oft von der Umweltbewegung geprägt (Benson 2020). Sie folgten zunächst einem Verständnis von Umwelt als nicht-menschlicher Natur, bis Anfang der 2000er Jahre eine stärkere Reflexion auf das Umweltkonzept selbst einsetzte. Doch auch viele rezente historische Ansätze betonen zwar den sozio-naturalen Charakter von Umwelten, widmen sich in ihrer Analyse jedoch vor allem dem „verkürzten“ Umweltbegriff (Brandt et al. 2019: 266), der Umwelt als von der Gesellschaft abtrennbaren Bereich darstellt. Diese thematische Fokussierung ist damit verbunden, dass sich viele wissenschafts- und technikhistorische Beiträge zu Umwelt primär mit naturwissenschaftlich orientierten Feldern beschäftigen. Im Vordergrund stehen entsprechend die Flora und Fauna, das Klima und natürliche Ressourcen (Oreskes & Conway 2010; Radkau 2018 [1987]; Isenberg 2005; Isenberg 2014). Dahingegen möchte das vorliegende *Special Issue* die wissenschaftshistorische Perspektive auf das Thema Umwelt dadurch erweitern, dass auch sozial-, human- und lebenswissenschaftliche Bereiche avisiert werden, etwa aus Psychologie, Pädagogik oder Hygiene. Als Formen der „Handlungswissenschaft“ (Hofer 2020) sind diese oft mit Alltagspraktiken und Fürsorgetätigkeiten verbunden und implizieren vergeschlechtlichte Konzeptionen von Umwelt.

Umwelt- und Geschlechterforschung überschneiden sich in Bezug auf ein jeweils zentrales Erkenntnisinteresse: Beide entwickeln eine kritische Perspektive auf dichotome Kategorisierungen und analysieren die problematische Trennung zwischen Außenwelt und Innenleben – zwischen Natur und Kultur, Arbeiten und Wohnen, Öffentlichkeit und Zuhause (MacGregror 2017; Mertlitsch 2019). In diesem Sinne lenkt der Fokus auf Umwelt und Geschlecht die Aufmerksamkeit auf „boundary work“ (Gieryn 1983) genauso wie auf die Berührungen des Innen und Außen, deren Komplementarität und gegenseitige Bedingtheit. Dies beinhaltet einen erweiterten Umweltbegriff, der gesellschaftliche Aspekte mit einbezieht. In den Mittelpunkt rückt damit die Bedeutung des Umweltdenkens für die Gestaltung sozialer Ordnungen diverser Skalierungen, von Uterus und Ei über das Bett im Schlaf- und Krankenzimmer bis zu familiären Bindungen.^3^

Schützende, bewahrende Idealbilder von Umwelt dienten und dienen der Legitimation traditioneller Geschlechterrollen. Ökofeministische Theoretiker*innen haben die Gemeinsamkeiten zwischen „der Gewalt, die Frauen angetan wird, und der Gewalt gegen die Natur“ in den Fokus gerückt (Merchant 1980; Keller 1983; Zitat: Mies & Shiva 2016 [1995]: 9). Die Befreiung der Frauen und der Natur, Umweltschutz und Feminismus sind aus dieser Sicht untrennbar verknüpft; zugleich wird die Figur der Frau als „natural environmental carer“ (Leach 2007: 68) unkritisch kolportiert. In pointiertem Unterschied dazu beleuchten die folgenden Beiträge die strukturellen Verbindungen zwischen zugeschriebener Umweltverantwortung und Ungleichheit der Geschlechter. Dabei verbinden sie die Analyse von Sorgearbeit und Reproduktionstätigkeiten mit der von Umwelt- und Milieuvorstellungen. Auf diese Weise schließt das *Special Issue *an bestehende Arbeiten am Schnittpunkt von Gender Studies und Wissenschaftsgeschichte bzw. -forschung an, die gezeigt haben, auf welche vielfältige Art und Weise sich kulturelle Normen und Geschlechterrollen in Vorstellungen der Natur einschrieben haben. Insbesondere die Naturalisierungsstrategien von Sorgetätigkeiten und das weibliche Naturbild der Naturgeschichte und -wissenschaft gerieten so in den Blick (Russett 1989; Schiebinger 1997; Sharp et al. 2018). Allerdings bleibt „die Natur“ auch in vielen der geschlechterhistorischen Analysen monolithisch und impliziert wird ein eng gefasster Umweltbegriff. Dagegen zeigen die hier versammelten Beiträge, dass einschlägige Wissensordnungen gerade nicht allein auf „die Natur“ rekurrieren. Vielmehr konzeptionalisieren sie sozio-biologische Umwelten, die stets an kulturelle Praktiken und Regulationstechniken gebunden sind.

Wir untersuchen den Zusammenhang zwischen Umwelt und Geschlecht, indem wir uns nicht nur Wissen aus Medizin und den Naturwissenschaften zuwenden, sondern auch sozial-, human- und lebenswissenschaftliche Bereiche einbeziehen. Mit der historischen Betrachtung heterogener Wissensfelder tragen wir bei zu einem jüngst aktualisierten Verständnis von Umwelt- und Umgebungswissen als einer zentralen „Kategorie des zeitgenössischen Denkens“ (Canguilhem 2009 [1952]: 233), die die modernen Wissenschaften disziplinübergreifend prägte, „ohne dass der Begriff dabei notwendigerweise im Zentrum stand oder überhaupt Verwendung fand“ (Wessely & Huber 2017: 12).^4^ Umgebungswissen war zentral für die Genese der modernen Wissenschaften vom Leben, die sich den „conditions d’existence“ (Georges Cuvier) zuwandten, von denen das Leben des Organismus’ abhing. So verstanden, beeinflusste und bestimmte die Umgebung die Formen und Funktionen eines Lebewesens nicht nur, sondern sie ermöglichte dessen Existenz.

Im Blick auf den Menschen war die Umwelt nicht nur Bedingung des Lebens selbst, sondern des Menschseins. Der frühmoderne Empirismus, der geschichtliche, ethnologische und staatstheoretische Ansätze verband (Pomata & Siraisi 2005), hatte die Lebensführung, Denkweise und Moral vergangener und fremder Völker und Kulturen aus klimatischen und geographischen Bedingungen erklärt. Es war, so ist anzunehmen, neben der Konjunktur der Umwelt in den Lebenswissenschaften auch diese Denktradition, aus der sich die tragende Rolle von Umgebung und Milieu für die Betrachtung und Analyse der modernen Gesellschaft speiste. In der literarischen Darstellung von Balzac bis Zola ebenso wie in geschichts- und kulturphilosophischer Lesart galt die Beziehung zur Umgebung und der Austausch mit dem „milieu correspondant“ (Auguste Comte) als Ausweis menschlich-zivilisatorischer Entwicklungshöhe (Bernard 1961 [1865]: 111–113; Sombart 1938: 14–15, 385–412; Sprenger 2014: 13–14). Diese Aufwertung der Umgebung schwingt auch in der im deutschen Kaiserreich aufkommenden Umweltschutzbewegung mit (Uekötter 2007), ebenso wie im Begriff der „Euthenics“, der sich um 1900 vorranging im englischsprachigen Raum verbreitete (Grandy 2006). Im Gegensatz zur anlagebezogenen Eugenik bezeichnet der Begriff die Vorstellung einer Verbesserung des Menschen durch die Verbesserung seiner Umwelt. Ähnliche Ideen bezeichnete im deutschen Sprachraum das Konzept des „sozialen Milieus“, das sich gerade in der Weimarer Republik verbreitete (Kabaum 2013). Es thematisierte die gesellschaftliche Bedingtheit von Gesundheit, Bildung und Erziehung und spiegelte sich in den umgebungsbezogenen Interventionsmaßnahmen der Sozialhygiene, Sozialpädagogik und Sozialmedizin.

Auf theoretischer und epistemischer Ebene waren im modernen Umweltbegriff Ambivalenzen zwischen Subjekt- und Objektstatus angelegt. Denn der Begriff war nicht nur von einem „Spannungsverhältnis zwischen Selbstbehauptung und Außendeterminierung“ geprägt, wie häufig kritisiert wurde (Spitzer 1942; Canguilhem 2009 [1952]; Zitat: Wessely & Huber 2017: 13). „Umwelt“ implizierte vielmehr auch die Verschiebung vom jeweiligen Gegenstand der Aufmerksamkeit auf sein Anderes: Der Organismus ist kein Teil seiner Umwelt, auch wenn diese seine Existenzgrundlage bildet (Pethes 2017: 139–141). Wie sich zeigt, ist diese Differenz geschlechtlich codiert. Indem die Analyse von *care *und reproduktiver Arbeit den Fokus vom umhüllten Subjekt auf sein feminisiertes Außen verschiebt, zeigt sie, auf welche Weise das Konzept der Umwelt eine Seinsweise und Existenzform vorschreibt: die des Umwelt-Seins. Wenn der Umweltbegriff einerseits zeigt „dass jedes Lebewesen ein Subjekt ist, das in einer Welt lebt, deren Mittelpunkt es bildet“ (von Uexküll & Kriszat 1970 [1934]: 11), so spricht er dabei zugleich denjenigen, die Umwelt her- und darstellen sollen, den Subjektstatus ab – ein Umstand, auf den insbesondere feministische Analysen von Schwangerschaft und Mutterschaft wiederholt verwiesen haben (Lyerly et al. 2009; Kukla 2010; Lupton 2012).

Mit dem Perspektivwechsel gerät die Herstellung und Gestaltung von Umwelten in den Blick. Im Mittelpunkt der hier vorgelegten Betrachtungen steht die Arbeit am Milieu und damit die ethischen, sozialen und ökonomischen Dimensionen der Kategorie Umwelt, die als geformte, humane Atmosphäre „genuin und immer politisch ist“ (Böhme 2013: 18). Dies ist für das Verständnis der Umwelt und ihrer Geschichte im weiteren Sinne relevant. Jüngere Studien zu Meeres- und Wasserbiologie sowie Aquarienkunde haben nicht nur auf den domestizierend-häuslichen Charakter dieses Milieuwissens verwiesen, das – ob im Labor oder im bürgerlichen Wohnzimmer – Teil der Beschäftigung mit dem Wohnen und dem Zuhause war (Kranz 2010; Lüttge 2017; Wessely 2013; Vennen 2018; Reiss 2020;). „[D]enn nichts anderes als die Lehre vom Haushalt oder das Wissen vom Heim bezeichnet die Zusammensetzung der griechischen Begriffe ‚oikos‘ und ‚logos‘“ (Wessely 2013: 38). Sie haben darüber hinaus in der Analyse der materiellen Forschungsausrüstung und semi-funktionaler Architekturen und Technologien mit großer Deutlichkeit den Aufwand, die Komplikationen und Frustrationen der Milieuerzeugung beleuchtet. Die „Umwelt“ verwandelt sich auf diese Weise von einer natürlichen Gegebenheit in eine historische Konzeption, deren Eigenschaften im Kontext spezifischer gesellschaftlicher Auseinandersetzungen bestimmt wurden. Das Milieu muss also hervorgebracht, eingerichtet und sorgsam gepflegt werden – die Umwelt ist das Ergebnis sozial situierter und kultureller Praktiken.

Reproduktionsarbeit, die Umwelten herstellt, ermöglicht und erhält, nennen wir hier „Cocooning“. So bezeichnete die Marktanalystin Faith Popcorn Anfang der 1990er Jahre den Rückzug aus Gesellschaft und Öffentlichkeit in das häusliche Privatleben als „die letzte kontrollierbare […] Umwelt“ (1991: 27). Kreiert im Licht sich individualisierenden Konsumverhaltens ebenso wie zur Beschreibung des „neuen Babybooms“, der Beförderung familiärer Domestizität und des Backlashs gegen die Frauenbewegung (Popcorn 28–29; vgl. Gasteiger 2008: 150–156; Faludi 2006), etablierte sich „Cocooning“ in den folgenden Jahrzehnten als ein Konzept postmoderner Gegenwartsdiagnose. Heute ist „Cocooning“ als schillernder Begriff in der Gesellschaftskritik ebenso wie in Innenarchitektur und Einrichtung verankert. Im Anschluss an seine alltägliche und kritische Relevanz verlängern die vorliegenden Beiträge den Blick zum einen in die Vergangenheit und verweisen auf die Wurzeln des „Cocooning“ als einer Kategorie und Praxis der bürgerlichen Gesellschaft seit dem 19. Jahrhundert (Mühlmann 1975: 180–188). Zugleich beschäftigen sie sich mit Praktiken der Umweltfürsorge in der Privatwohnung genauso wie im öffentlichen Radio und im Krankenzimmer, analysieren die bezahlte ebenso wie die unbezahlte Arbeit an der Atmosphäre. Grundlegend ist dabei ein Wechsel der Blickrichtung, der mit dem Prozessbegriff des Cocoonings einhergeht: vom viel diskutierten, oft gescholtenen „komfortbeschwichtigte[n]“ Individuum zur weniger beachteten Seite der Erzeugung von Komfort, Sicherheit und Intimität (Mitscherlich 1983: 396; Mühlmann 1975: 202–203).

Unter dem Stichwort des Cocooning wird die Produktion von Umwelten in einem umfassenden Sinne untersucht: Wie strukturierten hygienische Gestaltungsregeln und Einrichtungstechniken den privaten Wohnraum des 19. Jahrhunderts? Welche Rolle spielte die Idee des „Nests“ in modernen medizinischen, biologischen und psychologischen Perspektiven auf Schwangerschaft und Geburt? Wie leiteten bindungs- und entwicklungspsychologische Theorien Frauen in der zweiten Hälfte des 20. Jahrhunderts dazu an, sich selbst als Umgebung ihrer Partner und Kinder zu verstehen? Auf welche Weise schließlich wird in Krankenhäusern des frühen 21. Jahrhunderts das Spannungsverhältnis zwischen Sterilität und Atmosphäre – im räumlichen ebenso wie emotionalen Sinne – verhandelt? In der Beantwortung dieser und weiterer Fragen bringen die vier Beiträge des *Special Issues* von Kira Jürjens, Lisa Malich, Susanne Schmidt und Käthe von Bose heterogenes Quellenmaterial, Forschungsstränge aus unterschiedlichen Bereichen, Disziplinen und Epochen und verschiedene methodische Herangehensweisen zusammen. Die Beiträge aus dem wissens-, wissenschafts- und medizinhistorischen Kontext verbinden Material aus den Bereichen des Akademischen, Populären und Politischen und zeigen deren gegenseitige Beeinflussung auf. Die in engerem Sinne wissenschaftshistorische Analyse erweist sich dabei als unmittelbar anschlussfähig für Analysen der Science and Technology Studies und der Wissensethnologie ebenso wie für Diskussionen der literaturwissenschaftlichen Wissenschaftsgeschichte. Dies eröffnet den Blick auf die Schnittstellen und Unterschiede vielfältiger Disziplinen, Praktiken und Technologien der Wissensproduktion. Es ermöglicht den vier Beiträgen, Fragestellungen und Erkenntnisse der Gender Studies in das Feld der Umweltgeschichte einzubringen und zugleich das Forschungsfeld zu Umwelt- und Milieuwissen für die Geschlechterforschung zu erschließen. Bestimmte Umwelten, so wird sich zeigen, sollten und sollen vor allem von Frauen hergestellt und durch sie repräsentiert werden.

## Footnotes

1 „[T]here seems to be at bottom an *Urgedanke *emanating from an *Urgefühl*, an *idée-mère* born with a man—a projection of the feeling of the child within its shell, protected as it is in its mother’s womb“ (Spitzer 1942: 199). Zu Ei und Eierschale, vgl. ebd.: 6, 11, 19. Für eine historische Analyse von Spitzers Positivismuskritik, vgl. Feuerhahn (2017: 21–28).

2 Zum Konzept des „milieu intérieur“ bei Claude Bernard, vgl. Holmes (1986); siehe auch, zu pränatalen Milieus: Arni (2018: 171–186); Nemec & Zimmer (2019); zu Umwelthygiene und Toxizität: Sellers (1997); Buell (1998); und für eine globalhistorische Perspektive auf Umwelt und Gesundheit: Bretelle-Establet et al. (2020).

3 Zentrales Charakteristikum von Konzepten der Umgebung ist, dass sie kein Maßstabsproblem kennen, sie eignen sich „für die Beschreibung der Umgebung von Zellen ebenso wie für die Charakterisierung ganzer Gesellschaften“ (Wessely & Huber 2017: 13).

4 Für begriffs- und ideengeschichtliche Darstellungen vgl. Spitzer (1942); Canguilhem (2009 [1952]); Wessely & Huber (2017); Pethes (2017). Zum Einfluss ökologischer Konzeptionen auf die wissenschaftsgeschichtliche Forschung, vgl. Güttler (2019).
